# The impact of steatosis assessment in imaging

**DOI:** 10.1590/0100-3984.2024.57.e3

**Published:** 2024-05-31

**Authors:** Hilton Muniz Leão Filho

**Affiliations:** 1 Radiologist at Hospital do Coração, São Paulo, SP, Brazil. Email: hiltonmlf@gmail.com

Fat accumulation within the liver (hepatic steatosis) has been increasingly recognized as playing a significant role in the development of liver disease. Its prevalence was estimated to be approximately 38% worldwide in the 2016–2019 period, corresponding to a 50% increase in comparison with the 25% estimated for the 1990–2006 period^(1)^. In Latin America alone, hepatic steatosis affects approximately 24% of the population^(2)^. Hepatic fat deposition is typically associated with metabolic syndrome, cardiovascular disease, and even diabetes, as well as with an increased risk of developing atherosclerosis^(3,4)^. These associations prompted a change in the nomenclature related to the spectrum of steatotic liver disease (SLD), with the subtype comprising fat deposition, metabolic syndrome, and alcohol usage now being designated metabolic-dysfunction-associated steatotic liver disease^(5)^. If steatosis goes untreated, it progresses to inflammatory changes (metabolic dysfunction-associated steatohepatitis) in approximately 20% of patients, and approximately 20% of those patients evolve to fibrosis or even cirrhosis, which is an independent risk factor for hepatocellular carcinoma^(6)^. The growth in awareness of SLD has been accompanied by an improvement in its characterization through imaging, magnetic resonance imaging (MRI) having proved especially useful^(7)^. The development of confounder-corrected chemical shift-encoded MRI (CSE-MRI) techniques made it possible to obtain an accurate map of the liver fat percentage in a single breath-hold, being more precise in objective analysis than histology^(8)^.

In an article recently published in **Radiologia Brasileira**^(9)^, Gupta et al. correlated proton density fat fraction (PDFF) values obtained with CSE-MRI and magnetic resonance spectroscopy (MRS) in a group of patients without known liver disease. An excellent correlation was demonstrated between MRS and PDFF, in a circular region of interest (ROI) in the right lobe and in the liver parenchyma as a whole. Their data support the use of this technique in practice, enabling an easier analysis with a much larger area of parenchyma than that obtained with MRS. Another relevant finding is the large proportion of patients classified as having SLD: 32.7% by spectroscopy; 43.6% by CSE-MRI in the liver parenchyma as a whole; and 30.9% by CSE-MRI in the right lobe ROI. This finding is concerning but matches the large estimated number of patients with SLD worldwide. A recent article, using data from more than 40,000 patients in the UK-Biobank, reported a slightly lower proportion of patients in whom SLD was diagnosed on the basis of MRI findings: 27%^(10)^.

Three important points should be considered, the first being the inclusion criteria. Approximately 71% of the patients in the Gupta et al.^(9)^ study were classified as overweight or obese, which clearly influences the results. Another point concerns the somewhat vague definition of patients “without known liver disease”. Given that liver abnormalities are predominantly asymptomatic, numerous patients could be affected without showing clinical manifestations. This is relevant for algorithm definitions in large populations, in order to establish the best cost-benefit strategy. Another point is the definition of a relevant cutoff point for steatosis. The correlation with histology might have been insufficient, because it was not defined on the basis of prognosis but for general classification, arbitrarily. Therefore, perhaps other values should be selected for better risk characterization and intervention strategies. Recently, some articles have employed a fat-fraction cutoff point of approximately 15%, showing increased risk in the group with greater steatosis^(11,12)^. Finally, the definition of the ROI positioning is quite relevant. Even though the study in question demonstrated low variability between methods, like others in the literature^(13,14)^, the degree of steatosis was found to be greater when the total liver volume was evaluated than when the circular ROI was used or when spectroscopy was employed.

In summary, the Gupta et al.^(9)^ article provides further evidence to support the use of the current methods for quantifying liver fat by MRI, showing how quick and accurate they are, in comparison with spectroscopy as the reference standard. Another important point is the significant proportion of patients with steatosis in a population without known liver disease, which draws attention to a large number of patients at risk for developing severe liver abnormalities and even cardiovascular problems, related to metabolic syndrome. This last point highlights the issue of the number of people to be evaluated. Although MRI is a robust technique, it is unlikely to be able to investigate an entire population at risk for SLD in a national screening strategy. In this context, the role of quantitative ultrasound, which is garnering interest and is a less costly alternative, must be highlighted^(15)^. There are other alternatives, such as the potentially simpler and portable point-of-care MRI, recently shown to provide good results in phantoms and patients^(16)^. Finally, there is also the potential of “opportunistic” computed tomography and MRI in evaluating hepatic findings (and other parameters), to try to predict future risk^(17)^.
